# Sensor Network Infrastructure for a Home Care Monitoring System

**DOI:** 10.3390/s140303833

**Published:** 2014-02-25

**Authors:** Filippo Palumbo, Jonas Ullberg, Ales Štimec, Francesco Furfari, Lars Karlsson, Silvia Coradeschi

**Affiliations:** 1 ISTI, National Research Council, Area della Ricerca CNR, via G. Moruzzi 1, Pisa 56124 , Italy; E-Mail: francesco.furfari@isti.cnr.it; 2 Computer Science Department, University of Pisa, Largo B. Pontecorvo, 3, Pisa 56127, Italy; 3 Centre for Applied Autonomous Sensor Systems, Örebro University, Örebro SE-701 82, Sweden; E-Mails: jonas.ullberg@oru.se (J.U.); lars.karlsson@oru.se (L.K.); 4 XLAB Research, XLAB d.o.o., Pot za Brdom 100, Ljubljana 1000, Slovenia; E-Mail: ales.stimec@xlab.si

**Keywords:** middleware, sensor architecture, ambient assisted living, monitoring, elderly

## Abstract

This paper presents the sensor network infrastructure for a home care system that allows long-term monitoring of physiological data and everyday activities. The aim of the proposed system is to allow the elderly to live longer in their home without compromising safety and ensuring the detection of health problems. The system offers the possibility of a virtual visit via a teleoperated robot. During the visit, physiological data and activities occurring during a period of time can be discussed. These data are collected from physiological sensors (e.g., temperature, blood pressure, glucose) and environmental sensors (e.g., motion, bed/chair occupancy, electrical usage). The system can also give alarms if sudden problems occur, like a fall, and warnings based on more long-term trends, such as the deterioration of health being detected. It has been implemented and tested in a test environment and has been deployed in six real homes for a year-long evaluation. The key contribution of the paper is the presentation of an implemented system for ambient assisted living (AAL) tested in a real environment, combining the acquisition of sensor data, a flexible and adaptable middleware compliant with the OSGistandard and a context recognition application. The system has been developed in a European project called GiraffPlus.

## 1. Introduction

Ambient assisted living (AAL) solutions are currently becoming more and more common and are quickly moving from research prototype to actual commercial application. A key factor for the success of such systems is the smooth, easy and reliable integration of all hardware components and the integration of the hardware components with the higher level of the system performing monitoring and reasoning on the data. This is especially of importance in home care monitoring, as the system needs to be deployed in a private home and, therefore, requires a quick installation, adaptability to changes in sensor configuration and reliability over long periods of time. The GiraffPlus project is an EU FP7-funded project in which we develop and thoroughly evaluate a complete system that collects daily behavioral and physiological data from distributed sensors, performs context recognition, a long-term trend analysis and presents the information via a personalized interface. GiraffPlus supports social interaction between primary users (elderly) and secondary users (formal and informal caregivers), thereby offering an immediate and obvious benefit, which makes the system attractive and worth using. The GiraffPlus system is named after one of its components: the Giraff telepresence robot. The robot uses a Skype-like interface, allowing caregivers to virtually visit an elderly person in the home. The GiraffPlus system also includes a network of sensors placed in the home. These include physiological sensors for, e.g., weight, blood pressure and pulse oximetry, and environmental sensors. Data from these sensors are stored in a database and processed by an advanced context recognition system, which uses constraint-based temporal reasoning in order to detect events on-line or perform inferences about long-term behaviors and trends. Secondary users can access the data and events remotely through personalized services: users may have access to different data, may be interested in different events and may want the information presented in different ways.

This paper focuses on the architecture of the GiraffPlus system and, in particular, the hardware components and their integration via a middleware infrastructure. It first presents related work, then an overview of the system, the hardware and software components (and, in particular, the middleware), the data storage, the context recognition and configuration planning. Finally, the paper reports on the testing of the system and presents the conclusions.

The key contribution of the paper is the presentation of an implemented system for ambient assisted living (AAL) tested in a real environment. It combines the acquisition of sensor data via a flexible and adaptable middleware with high-level reasoning and, in particular, context recognition.

## 2. Related Work

The increase in lifestyle related diseases, together with an aging population, are important driving forces to develop systems that facilitate the monitoring of health status independent of location: at home, at work or in the hospital. One important trend is that both the acquisition and processing of physiological data are moving out from the hospitals, allowing patients to stay at home or at work. Furthermore, there is a trend towards technologies that achieve prevention of disease. These technologies have now reached the point where they have become enabling technologies in clinical applications [1]. Substituting traditional sensors by smart textiles for health monitoring has been another hot topic in the last few years [2]. Several research groups and industry have initiated projects in this area, for instance MyHeart (http://www.hitech-projects.com/euprojects/myheart/), an EU FP6 project with Philips Research as one of the major contributors, focusing on vital sign monitoring, and HeartCycle (http://heartcycle.med.auth.gr/), also coordinated by Philips, produced a key result in the provision of closed-loop disease management. In the industry, the Continua Health Alliance [3], which started in 2006 to enable an interoperable personal telehealth ecosystem, has become a major force in the personal telehealth domain, and over a dozen interoperable products have already been certified. Continua has also made great progress in defining interoperability for the LANand WANinterfaces and now enables end-to-end interoperability. For the transport level, Continua has recently adopted the ZigBee Health Care Profile (http://www.zigbee.org/) and the Continua Bluetooth Profile in addition to Bluetooth Low Energy (BLE) and USBand the ISO/IEEE11073 Personal Health Device family of standards [4] for the data level.

In the GiraffPlus project, we aim at deploying a number of sensor devices that are pervasively integrated in the home or that can be used by the elderly to collect vital signs measurements. The difference with the end-to-end architecture of Continua is that we are interested in a holistic view of the home environment, and we aim to collect information potentially coming from devices belonging to different application domains: personal health devices, home automation devices, entertainment devices, smart energy management devices, and so on. The novelty of our approach is in the integration of existing sensors into one system aimed at providing increased home safety and in supporting monitoring physiological parameters over a long period of time. The acquisition of physiological data will include the reliability of non-invasive data acquisition methods and the reliable detection and filtering of measurement artifacts. In wireless sensor networks (WSNs), data aggregation, fusion and filtering are an important topic and combined with aspects, such as security, robustness, reliability and extended lifetime, are very challenging. The level of complexity increases even more considering that sensed data may be originated by subsystems having different constraints and using different protocols. For example, user location can be derived by using hybrid systems [5] based on infrared vision, a radio tag worn by the user and by a number of sensor nodes placed in the environment.

To address these issues, a component-based framework has been developed in the GiraffPlus project. In this framework, different components can be deployed to accomplish its relative task. The aggregation, fusion and filtering of data coming from the WSNs are done in specific components called gateways, which, being aware of the particular technology below them, collect and aggregate sensed data and present it in a common format for all the applications in a transparent way. The context recognition component, for example, can use these data without worrying about the particular communication protocol needed to gather them. In the same way, a specific component offers a secure and reliable channel to gather and store data, hiding all the complexity of handshaking and the replica mechanism to the application components in the common APIexposed by the low level layers (*connectors*) to the upper layers (*applications*).

In this context, the integration of all components is done by means of a distributed middleware, which is able to hide the heterogeneity of the sensor devices. This kind of architecture, composed of different layers that communicate through a middleware layer, is becoming increasingly important [6]. Since 1997, the OSGiplatform has attracted much attention among the middleware technologies, thanks to the modularity layer it adds on top of the Java programming language and the service-oriented architecture (SOA) adopted to dynamically bind service components. It has been recognized as the Java middleware, and it became mainstream in 2003, when big players, like IBM and Oracle, started to adopt OSGi as the foundation run-time for enterprise applications (i.e., Eclipse). In the academic world, the SOCAMarchitecture [7] was one of the first to suggest an OSGi-based infrastructure for context-aware applications. In fact, OSGi is suitable for acting as a smart gateway able to link different network technologies, but still providing the easy extendability and configurability of the system, which are valuable properties to meet the interoperability requirements of dynamic environments. For these reasons, OSGi has been chosen as a reference framework in the development of the middleware solution integrated in the GiraffPlus project.

## 3. Overview of the GiraffPlus System

**Figure 1.**
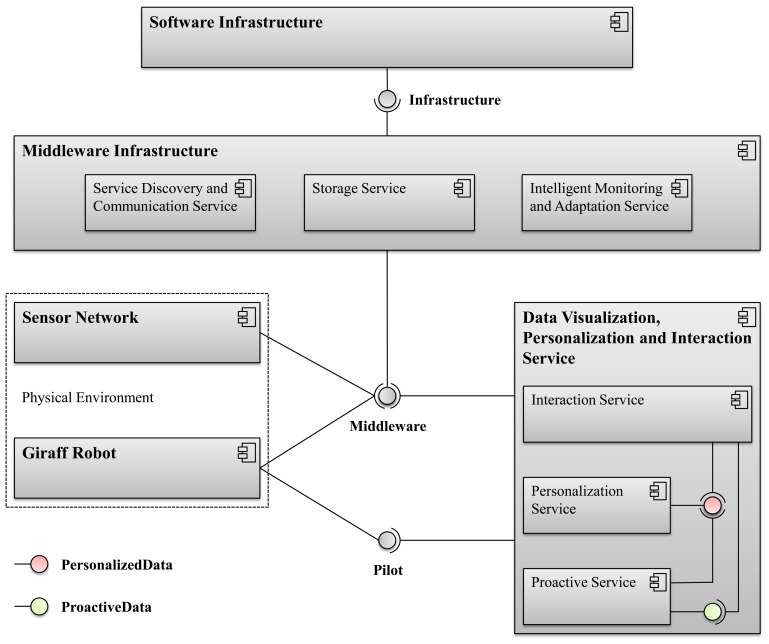
The GiraffPlus system architecture.

This section describes the overall architecture of the GiraffPlus system. Figure 1 shows the general component diagram of the GiraffPlus system. In particular, three main components have been identified: (a) *Physical environment and software infrastructure*; (b) *Data visualization, personalization and interaction service*; (c) *Middleware infrastructure*.

The physical environment and software infrastructure component represents the basic level of the functionalities of the GiraffPlus system. All the data services are grounded on functionalities of this part of the system. This module is also in charge of providing the common and interoperable communication service. In particular, the:

- The software infrastructure component includes information management software, storage management software, IT operations management and security software and other infrastructure software. This component provides data integration for achieving the consistent access and delivery of data across the spectrum of data subject areas and data structure types in the system to meet the data consumption requirements of all the application and business processes of the GiraffPlus services.
- The physical environment component includes the sensor network and the Giraff robot. The role of the sensor network is to gather the data generated by the sensors (deployed in the elderly person's home), as well as to provide the rest of the system with the (possibly pre-processed) collected data. The Giraff robot component implements the GiraffPlus tele-presence functionalities, also providing a pilot service to remotely control the robot.

The data visualization, personalization and interaction Service component is the part of the system responsible for creating user-oriented service. A broad way to summarize the module is to provide different end-users with suitable interaction modalities for the available services [8]. The module is subdivided into a front-end part, the interaction service and a back-end part based on two basic services, the personalization and the proactive services:

- The interaction service is the basic front-end of GiraffPlus to all the human users' contacts. It will provide visualization services adapted to the different interactions created within the system. Depending on the classes of end-users, personalized services and specific dialogue boxes are offered, which take into account his/her specific needs/roles.
- The personalization service acts as the back-end of the interaction service and is in charge of collecting and keeping up-to-date all the data needed to generate personalized interactions. It basically creates and dynamically maintains profiles for all the end users involved in GiraffPlus and also provides some reasoning services specifically tailored for the persons involved (e.g., the reminder settings and associated dialogues).
- The proactive services are responsible for collecting specific functionalities to prepare content to be sent from the technological modules to users. Examples of GiraffPlus proactive services can be the reminder and the warning report builder.

Finally, the interconnections among components are also relevant. In this regard, a crucial role is played by the middleware infrastructure component, as it provides the central connection point that is shared by all the components according to the needed information exchanges. It provides to the data visualization, personalization and interaction service (DVPIS) and to any other advanced service to be integrated in the GiraffPlus system a common way to interact with the key functionalities of the system. In particular:

- The service discovery and communication service, providing the primary functions to establish and maintain connections between nodes of the GiraffPlus ecosystem. It enables different services on different nodes to share, modify and manipulate data.
- The storage service, acting as a proxy for accessing the storage management functionalities deployed remotely. Thanks to this service, a generic service built upon the middleware can access historical data and configurations stored on the database without caring about connectivity and how to access it.
- The intelligent monitoring and adaptation service act as a proxy for accessing the context/activity recognition and configuration functionalities deployed on a dedicated server. As for the storage service, this component is necessary to avoid that other high level components must be aware of connectivity and access settings.

The entire data and workflow in the GiraffPlus system uses the middleware infrastructure to realize a distributed assistive system. For instance, the sensor network provides the data storage with the data collected through the sensors and that need to be placed in the database; the context inference system retrieves the sensors data from the data storage and exploits them in order to reason about the person/ambient status; the configuration planning system selects the set of sensors needed to implement a requested monitoring activity. The personalization service dynamically provides user profiles that are stored in the database. All these actions are done interacting with the middleware infrastructure that enables independently designed applications, software components or services to work together, supporting data consistency, composite application and multi-step process styles of integration by connecting various, heterogeneous devices and services to a single, unified network.

Part of the system is implemented using a cloud computing solution. There is a centralized database, and some services (intelligent monitoring and adaptation service) are running on the GiraffPlus cloud infrastructure and not locally in elderly person's home. These modules are represented in Figure 1 as part of the software infrastructure component.

## 4. Hardware Components

In the following section, we briefly describe the most important components used in the system. We first consider the sensors that are used for monitoring physiological parameters, then the sensors for monitoring activities and behaviors and, finally, the sensors used to alert emergency situations. The sensors have been selected according to the results of a user requirement study involving both primary and secondary users that have identified the activities and data that are important to monitor. The Giraff robot is also presented.

### 4.1. Monitoring of Physiological Parameters

The GiraffPlus system includes sensors for blood pressure monitoring, glucose level measurements, temperature measurements, weight and oxygen saturation, which are provided by Intellicare (http://www.isasensing.com/). These devices use Bluetooth as the transport layer.

- The blood pressure monitor has a cuff that should be placed around the upper arm. The measurement is performed automatically, and the system provides automatic averaging of three measurements within two minutes and detects pulse irregularities during the measurements.
- The glucose meter is recommended to be used before and after a meal by patients with diabetes, to control the glucose level and adjust the insulin injection. The system uses a small sample volume of blood (0.5 l), and the blood sample is performed by the use of a so-called safe strip injection, which avoids hand contact with the test strip. The test is received within 5 s.
- The thermometer measures the temperature in the ear and delivers a result in 1 s.
- By the weight scale, changes in weight can be monitored. This is an important warning for patients with heart failure as a sign of increased body water and load for the heart.
- The pulse oximeter available is called NONINOnyxII. The device is certified as compliant with the Continua™ Version One Design Guidelines. It connects via Bluetooth 2.0 wireless technology and can connect to communication devices (cell phones, PDAs, PCs, *etc.*). It is designed to meet the requirements of the emerging open standards, such as the Bluetooth Health Device Profile (HDP), IEEE11073 and Continua, and it is equipped with memory for storing up to 20 measurements and algorithms for selecting measurements.

### 4.2. Behavior and Activity Monitoring

The sensors used for behavioral and activity monitoring are provided by Tunstall (http://www.tunstall.co.uk/). FAST passive infrared motion detectors (PIR), electrical usage sensor, body fluid Sensors and a universal sensor that can be configured according to different needs (door usage, bed/chair occupancy) have been used in this work:

- Typically, several FAST passive infrared motion detectors (PIR) are placed in the bedroom, the bathroom and other rooms according to needs. From these sensors, information about the time spent in different rooms can be monitored.
- The electrical usage sensor is plugged in between an electrical apparatus, such as a water boiler, and provides information about how often the apparatus is used.
- Door usage can be monitored by the universal sensor. Both the cases where the door normally is open and normally closed can be monitored; also, internal doors, such as a bedroom door, or the door of a refrigerator can be monitored.
- Using the bed/chair occupancy sensor, the presence of a person can be determined. The sensor is a pressure sensitive mat and detects pressure/no pressure and requires a universal sensor. In combination with a PIR, it can report more complex activity behavior (the bed sensor turns off, the bathroom PIR turns on, *etc.*).
- The body fluid sensors (enuresis sensors) should be placed between the mattress and sheet and provide a warning upon the detection of moisture.

### 4.3. Emergency Situation Detection

A number of emergency situation detection sensors have been used, in particular the telecare flood detector, wireless carbon monoxide detector, wireless smoke detector and wireless heat detector provided by Tunstall:

- The telecare flood detector provides an early warning of flood situations, such as taps being left on. It should be placed on a flat surface close to a bath, wash basin, toilet or sink.
- The wireless carbon monoxide detector provides warnings in the case of dangerous CO levels.
- The natural gas detector provides an early warning of dangerous levels of gas. It can be linked to the gas shut off valve to automatically cut the gas supply off, if a leak is detected.
- The wireless smoke detector alarms if smoke is detected and also provides auto low battery reporting.
- The wireless heat detector provides additional protection against the risk of fires in rooms where smoke detectors are unsuitable, e.g., the kitchen. The detector raises an alarm when the temperature reaches between 54°C and 62°C.
- The temperature extremes sensor monitors for low and high temperature extremes in addition to the rate of rise in temperature. The sensor helps to minimize the risks associated with changes in temperature.
- Two types of fall sensors are used, either worn around the wrist or waist. The sensors raise an alarm in the case of a fall. If normal activity is detected after the fall, the alarm can be canceled.

These sensors, like the ones used for behavior and activity monitoring, operate on the European social alarm frequency 869 MHz.

### 4.4. The Giraff Robotic Platform

**Figure 2.**
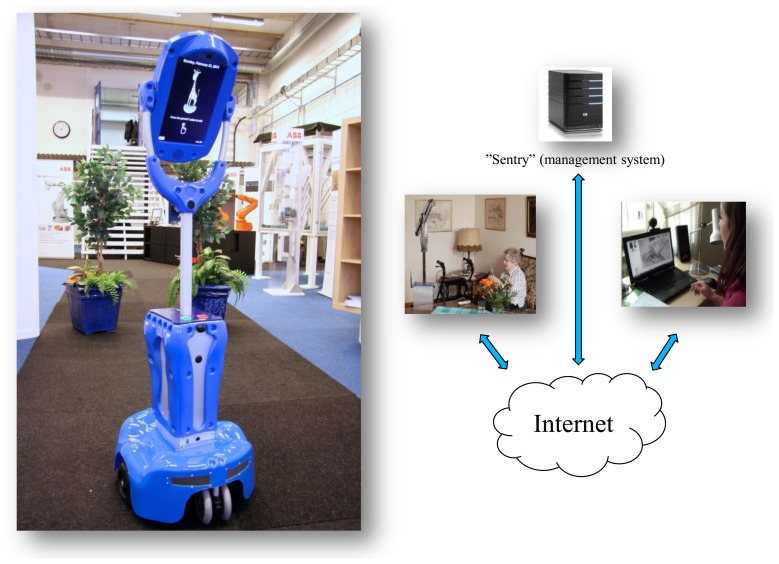
The Giraff platform.

The Giraff platform has three main architectural components (Figure 2):

1. The “Giraff” is a remotely controlled mobile, human-height physical avatar integrated with a videoconferencing system (including a camera, display, speaker and microphone). It is powered by motors that can propel and turn the device in any direction, even backwards. The Giraff is placed in a home or care facility and allows a caregiver (formal or informal) to virtually visit the residents there, move about and freely interact with them (talk and listen, see and be seen) just as if that caregiver were physically present.
2. The Giraff is accessed and controlled over a standard Internet connection via the “pilot” computer/laptop client. From a remote location, a person with no prior computer training can “visit” a home and intuitively navigate the Giraff down hallways, through doorways and around tables and chairs. Visitors can also look around via a pan/tilt/zoom camera and be seen and heard in real time via a life-sized portrait image from their webcam.
3. Care organizations manage Giraffs and users via “Sentry”, a user management policy and supporting administration database that ensures only caregivers authorized by the resident can connect to the home and only under the circumstances (day, time, *etc.*) dictated by the resident. Some trusted caregivers may be allowed in certain situations to connect without the call being answered by the resident.

## 5. The Middleware

GiraffPlus is a set of software modules that helps to build so-called “assistive systems” by connecting various, heterogeneous technical devices into a single, unified network. In GiraffPlus, a single device that is connected to the system is referred to as a “node”. There are two ways to integrate a device into the GiraffPlus system, assuming that the device in question is networked (can send and receive data using a network protocol, either wired or wireless). The first way is to install a specific piece of the GiraffPlus platform on the device, the so-called “middleware” (as in the case of the Giraff robot). The middleware software contains the communication infrastructure of the GiraffPlus platform, and all devices that run the middleware can actively participate in the communication of the system. The second way of connecting devices to the GiraffPlus system does not require a given device to run the GiraffPlus middleware. The device in question is rather connected to a node that runs the middleware, and this node is used as an intermediary by the system in order to control the additional device. For many devices, such as low-power wireless sensors, this is the only possible way of connecting them to the system, simply because they cannot run any additional software beyond their firmware. Although these slave-devices cannot actively participate in the communication with the rest of the system (as they are just queried for data), their advantage over regular nodes is that they can simply be “plug-and-played” into a running system.

The GiraffPlus middleware is inspired by the concrete architecture of the UniversAAL [9] middleware and partially derived from the PERSONA [10] project. The UniversAAL (http://universaal.org/) project is the most recent European initiative aimed at delivering a software platform for AAL, which started to consolidate the architecture and software developed in recent research projects, like PERSONA http://www.aal-persona.org/, SOPRANOhttp://www.soprano-ip.org/), OASIS(http://oasis-project.eu/), and mPOWER(http://www.sintef.no/Projectweb/MPOWER/). One of the major tenets of the UniversAAL approach is to separate the reference architecture used to lay out the smart-home environment from the concrete architecture and implementation used to realize the intelligent services. This abstraction allows the design of different platforms, for example the ones based on different paradigms and technology, but still sharing common building blocks. In this way, common building blocks enable the creation of an ecosystem by ensuring the interoperability at the level of the interface or functional services. One of the main goals of the GiraffPlus middleware is to be compatible at the level of reference architecture with UniversAAL-based systems. This means that with some simple adapter, the components used in GiraffPlus will be able to run in a UniversAAL-based system.

Besides the middleware, the GiraffPlus platform is made up of various other software parts, which we will refer to as “higher level components”. The middleware is the basis for all these higher level components. Components rely upon it, as the middleware is capable of hiding the distribution and heterogeneity of the diverse devices that make up the system. From an application programmer's perspective, this means that he does not need to worry about the fact that some of the applications (and higher level platform components) involved in the workflow may actually be running on other devices. The application simply forwards all of its messages and requests to the middleware component, which makes sure that each message reaches its recipient. The middleware does, of course, not hinder any application from accessing a device's operating system API. It simply eliminates the need for resolving dependencies.

**Figure 3.**
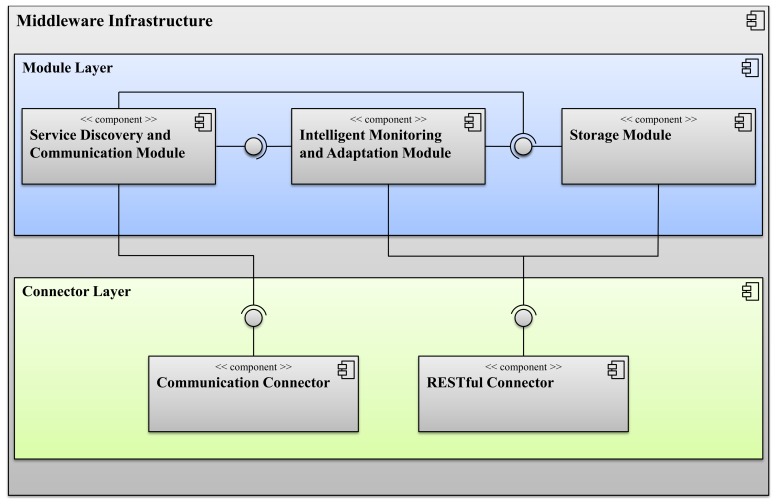
An in-depth view of the middleware component.

Hiding the distribution and heterogeneity of the devices is actually just a part of the middleware's job. The middleware infrastructure is composed by different modules and connectors offering APIs to access the main functionalities of the GiraffPlus system. Its architecture presents two main layers implemented as a set of OSGi bundles: a connector layer and a module layer. The architecture of the middleware infrastructure is reported in Figure 3.

The connector layer provides a pluggable mechanism in order to enable communication capabilities among nodes and the possibility to access remote resources via RESTful (representational state transfer) interfaces. The connector layer is a set of artifacts that make use of third party libraries. Some notable examples are MQTTfor the communication connector and Jersey for the RESTful connector.

The module layer is designed in order to detach the connectors from the rest of the architecture by providing a connector-independent set of APIs. The GiraffPlus middleware implements a specific module for the service discovery and communication, intelligent monitoring and adaptation and storage capabilities:

- The service discovery and communication module is responsible for ensuring that communication can take place across all the networked nodes. To do this, nodes must firstly be discovered and enabled to exchange messages. The communication building block defines how data is transported from the sender to the receivers. For the data exchange between the communicating parties, different communication modes (publish/subscribe and REST methods invocation) may be supported. Additionally, possible roles that are involved in the communication (e.g., publisher and subscriber) are specified.
- The storage module is responsible for providing a general database service for all the data generated by parts of the system and providing data access functionalities. Specifically, the role of this component is to manage a database containing all the data collected through the middleware service and generated by other system's components (for instance, the sensor network). Additionally, it enables other components to access the information and reason about it (also considering historical evolution). This module guarantees permanent data to be gathered, as well as offering the general possibility to use the future long-term experimentation as a benchmark gathering.
- The intelligent monitoring and adaptation module is the component responsible for context/activity recognition and configuration planning. This part of the system encompasses two general reasoning systems, namely:
  – The context inference system, which is in charge of implementing the requested monitoring activities by means of context/activity recognition and relies on a timeline-based representation of the data generated by the sensors.
  – The configuration planning system, which is responsible for providing suitable configuration settings for the sensor network according to the requested monitoring activities.

The details about the middleware interfaces and the functionalities of each module are illustrated in the following sections.

## 6. Service Discovery and Communication

Within the proposed AAL ecosystem, an AAL space is intended to be the physical environment (such as the home of an assisted person) in which independent living services are provided to people that need any sort of assistance. In such a virtual ecosystem, hardware, as well as software components can “live” while being able to share their capabilities. In this space, the proposed platform facilitates the sharing of three types of capabilities: service (description and discovery of components), context (the database on shared models) and control (the state of the component of the system). Therefore, connecting components to the platform is equivalent to using the brokerage mechanism of the middleware in these areas for interacting with each other. Such connectors together with the application logic behind the connected component are called altogether AAL services [11], as at its core, an assistive system is nothing else, but a local private network of technical devices. When additional nodes try to (re)join the system, the discovery mechanisms of the middleware automatically recognize and integrate all qualified nodes within reach. The GiraffPlus middleware has different mediators to discover nodes and reach them, and each of them is responsible for the delivery of a certain message category: the buses. On these buses, a listen-announce protocol has been implemented to let nodes be discovered. A publish-subscribe pattern is used to send and receive messages.

### 6.1. The Buses

The service discovery and communication functionalities are realized by intelligent buses, namely the context, service and control buses. All communications between services can happen in a round-about way via one of them, even if, physically, the services are located on the same hardware node. Each of the buses handles a specific type of message/request and is realized by different kinds of topics. Topics are realized exploiting the communication connector interface based on the MQTT (http://mqtt.org/) protocol. MQTT is a machine-to-machine (M2M) connectivity protocol designed as an extremely lightweight publish/subscribe messaging transport [12].

A GiraffPlus node can discover and be discovered by other nodes publishing and subscribing to topics belonging to the service bus. A generic service bus URIscheme (topic) has the following format:

≪location≫/serviceBus/≪serviceURI≫

where location identifies the house of the assisted person, serviceBus is the keyword that identifies the topic as a service bus topic and serviceURI is the unique identifier of the service with its path (i.e., sensor/pressure/kitchen/chair/sensor-01).

A service can publish and retrieve context information using the context bus. Context bus topics have the same format of service bus topics, except for the keyword used to identify topics:

≪location≫/contextBus/≪serviceURI≫

The system provides a dedicated set of topics to publish and retrieve the “health status” of service or system information (i.e., a heartbeat monitor or permissions attached to a service). These topics are grouped under the control bus root:

≪location≫/controleBus/≪serviceURI≫

**Figure 4.**
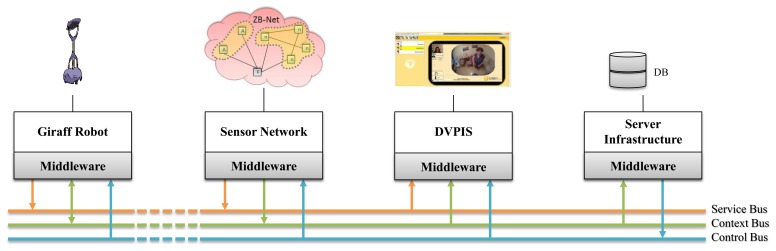
Interaction between components and buses. DVPIS, data visualization, personalization and interaction service.

Figure 4 shows a typical usage scenario of the buses. The Giraff robot announces its presence on the service bus, collects and publishes data (i.e., positions) to the context bus and retrieves the status of the system from the control bus. The sensor network only publishes environmental data (i.e., temperature, pressure, presence). The data visualization, personalization and interaction service (DVPIS) discovers the services in the house and retrieves real-time sensory data (i.e., it displays to the user which sensors are installed in a particular room and its current readings). The server infrastructure listens to all sensors readings (i.e., to store the data in the database) and publishes information about the status of the server components (i.e., connectivity issues).

### 6.2. The Listen-Announce Protocol

The middleware is in charge of presenting the available sensors and services in the system implementing a listen-announce mechanism on the service bus. The resources are presented with a message on the relative topic in the service bus. The message is a JSON-formatted (http://www.json.org/) descriptor document called *ServiceDescriptor*:
{ “id”:“56a2fc89”, “category”:“sensor”, “messageFormat” :[{“unit”:“degC”, “name”:“temp”}], “type”:“Temperature Sensor”, “recipient”:[], “contextBusTopic”:“sensor/56a2fc89”, “serviceBusTopic”:“sensor/56a2fc89”, “URI”:“sensor/56a2fc89”, “room”:“kitchen” }

containing the ID associated with the service, the category of the service (a sensor in the example), the message format field indicating the unit or the format of the value to be published, a type (i.e., a reminder service or a temperature sensor), a recipient list field (an empty set means everybody), the topics where the information and messages will be published, a URI for the RESTful interface and the room where the sensor is installed (null for pervasive services).

**Figure 5.**
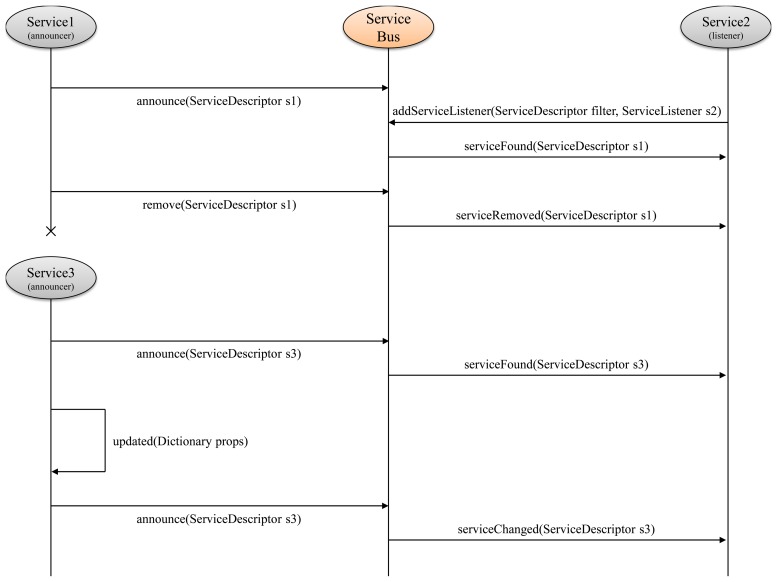
The announce-listen protocol model.

Once a resource has been announced on the service bus, a generic service can search for it, filtering on the descriptor fields (i.e., on the category or the room, and so on), and use it. The sample sequence diagram in Figure 5 shows the interaction between services in the discovery phase. A generic service announces its presence using the service discovery and communication module API. The module publishes a message on the service bus that contains the *ServiceDescriptor* of the service, serialized as a JSON document. These messages are *retained*, exploiting the functionality of the MQTT protocol that records the latest value of a topic. New subscribers to retained topics immediately receive the most recent value. All the service bus topics are retained, so when the GiraffPlus middleware starts, it can forward the ServiceDescriptor to services that add their listener after an announce is made by a service running on another middleware instance. When a service wants to use another service that meets a set of properties, it defines a ServiceDescriptorfilter, and it queries the middleware providing a listener. If a service that satisfies the requests is already present or became available, the service discovery and communication module invokes the listener's callback, passing the relative descriptor as an argument. Now, the listener can begin to subscribe to the right topics (listed in the descriptors). A service listener has different callbacks to be notified when a service is found, changed or removed.

### 6.3. The Publish-Subscribe Pattern

The aim of the middleware is to provide a publish-subscribe mechanism for accessing the context information about the physical environment, physiological data and system information. The principle of the publish-subscribe communication model is that components that are interested in consuming certain information register their interest (subscribers). Components that want to produce certain information do so by publishing their information (publishers). The middleware takes care of dispatching information by means of buses. Any service interested in monitoring these data can subscribe to the relative service, context and control bus topics using the middleware API.

There are three principal types of publish-subscribe systems: topic-based, type-based and content-based [13]. With topic-based systems, the list of topics is usually known in advance. In type-based systems, a subscriber states the type of data in which it is interested. In the content-based systems, the subscriber describes the content of messages it wants to receive. The GiraffPlus system tries to put together the benefits from the different types using the information provided in the announce phase of the services. When a listener receive a set of descriptors that matches with the filter used, it can refine further its search and than subscribe only on the topics listed in the descriptor of the desired services. A service can also subscribe only to topics that belong to a particular type: service, context or control bus topics.

Once a service has been notified by the middleware that a requested resource is available with its *ServiceDescriptor*, it can fetch from it the relative context bus topic and can subscribe to it starting receiving messages. Each message that flows on the context bus topics has a standard message format that reflects what is declared in the descriptor with the addition of the source ID and a timestamp:
{ “id”:“56a2fc89”, “timestamp”:“2014.01.13.13.50.57+0100”, “values”:{“temp”:“18.5”}} }

## 7. The Mobile Middleware

**Figure 6.**
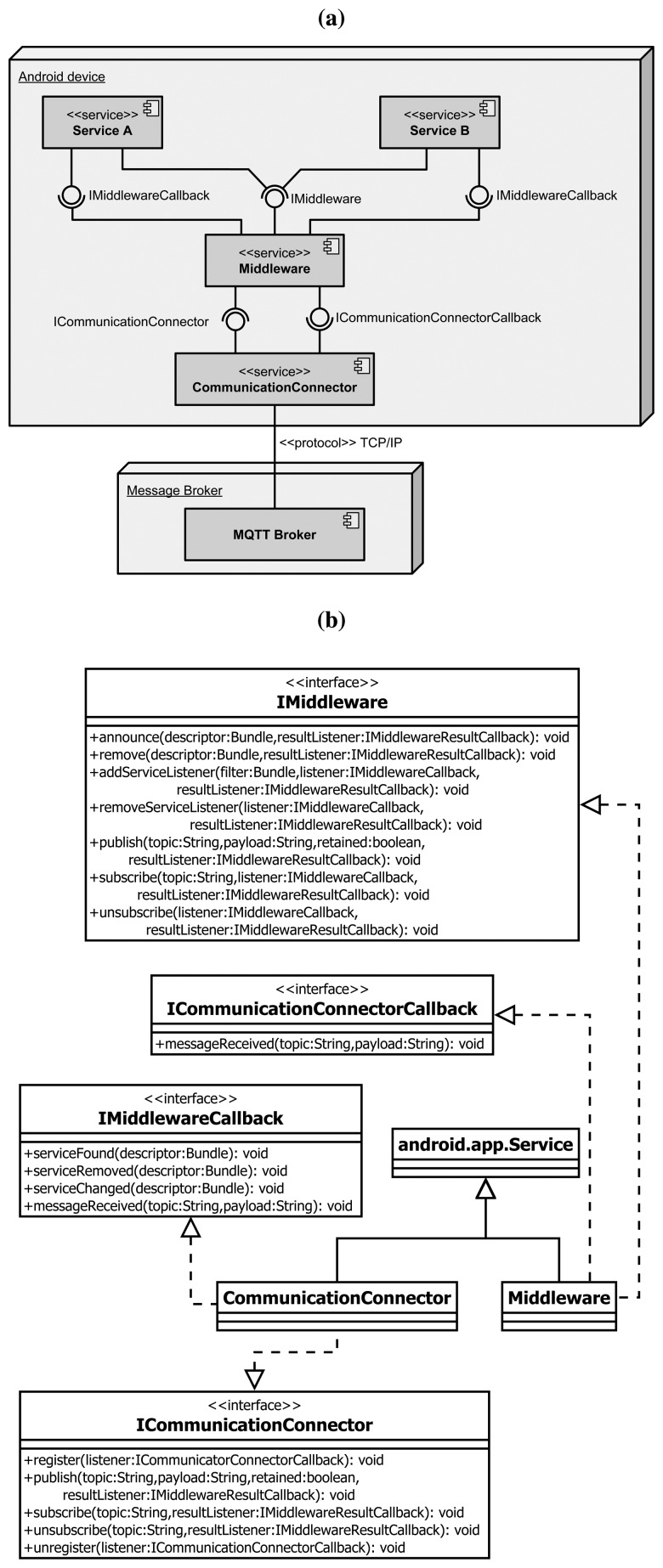
The Android middleware architecture with component (**a**) and class (**b**) diagram.

In order to collect and aggregate data from physiological sensors (Section 4.1) and to give the primary user a personal device connected to the GiraffPlus network, a mobile version of the middleware running on Android platforms has been developed. The mobile middleware has been designed aiming at optimizing both the performances and the modularity to reflect the OSGi-based reference architecture. It is made of two components running as services in the background and providing the required functionalities on demand. These two components, shown in Figure 6a, are the middleware and the CommunicatorConnector. On top of them, several services (Android applications) can be created to send or receive sensor data through the network by interacting with other devices.

The scope of the middleware component is to provide to the upper level applications a common interface, compliant with the OSGi version of the middleware, for discovering other sensors/services in the network, subscribing in order to receive their updates as consumers or publishing data as producers. The CommunicatorConnectorcomponent is used to decouple the business logic contained at the middleware level (handling the control and context information) from the actual mechanism used to exchange messages in the network. This module is in charge of dispatching and delivering the messages based on a given communication protocol.

To guarantee the same level of abstraction between the middleware modules, both the middleware and the CommunicationConnectorcomponents are implemented as bounded services, a particular type of service that exists only when serving another application. The inter-process communications among the components are performed using a well-specified interface defined with the Android Interface Definition Language (AIDL). The AIDL interface definition has been used to handle multithreading, which means potentially concurrent IPCson the same service.

In the proposed system, the IMiddleware interface (Figure 6b) offers the same set of functionalities to the upper level applications of the OSGi version, allowing a GiraffPlus component to be easily ported to the mobile platform.

## 8. Data Storage

The guiding principles in the design of the GiraffPlus Long-term Data Storage (LTS) system have been security, flexibility, reliability, efficiency and scalability. Security is paramount, as we are dealing with sensitive personal health information. Flexibility is important, as GiraffPlus is a research project, and it is impossible to foresee all the possible data structures that might, at some point, be stored in the system, as well as all possible connections between them. At present, two types of data are stored in the database: the home configuration data, which is needed for middleware auto-configuration, and sensor data from all sensors in all GiraffPlus-enabled homes. The storage system needs to be able to reliably store vast amounts of sensor data and enable users and other GiraffPlus components to efficiently access the stored data. Last, but not least, the system needs to be scalable, as we expect the amount of data to vastly increase during the course of the project and after, during the commercialization phase of the project.

Apart from the GiraffPlus middleware storage module, which enables secure access to stored data via the middleware, the GiraffPlus Long-term Data Storage system is composed of four components:

- the database, which stores the actual data,
- the GiraffPlus MQTT listener component, which is connected to the central MQTT broker and forwards all relevant data to the database in a secure fashion,
- the GiraffPlus LTS RESTful web service, which enables secure access to the database,
- the GiraffPlus engineer GUI, which enables GiraffPlus professionals to enter configuration data for individual homes,
- the GiraffPlus Certificate Agency, which issues certificates used to authenticate various users and components in the GiraffPlus ecosystem.

### 8.1. The GiraffPlus Database

Following the guiding principles listed in the previous section, we selected the MongoDB (http://www.mongodb.org/) database to serve as the basis of the GiraffPlus LTS, which is a widely used, open source NoSQLdocument-oriented database system developed and supported by 10gen (http://www.10gen.com/). We make heavy use of its replication feature, which replicates data over specified groups of servers, increasing data reliability, as well as efficiency. In case one of the replica servers fails, data can still be retrieved from other replicas, while the broken server is being replaced. We also use the sharding feature, which distributes the data over many replica groups, ensuring horizontal scalability of the system and increasing efficiency via load-balancing. The NoSQL nature of the system ensures the required flexibility of the system, since the database stores collections of generic JSON objects, which can represent any data structures that might come up during the lifetime of the project.

### 8.2. The GiraffPlus MQTT Listener

The GiraffPlus MQTT Listener is a standalone component, which connects to the central GiraffPlus MQTT broker, listens to all incoming sensor messages and passes the sensor data to the GiraffPlus LTS database in a secure fashion. It was implemented with simplicity and efficiency in mind, as it needs to be able to store large volumes of incoming sensor data from all GiraffPlus-enabled homes.

### 8.3. The RESTful GiraffPlus Storage Web Service

The GiraffPlus LTS RESTful (representational state transfer) web service enables efficient access to the data stored in the database. It is implemented in Java using the Jersey framework (http://jersey.java.net/), which is an open-source, production quality, JAX-RSreference implementation for building web services. To run the web service, we use Apache Tomcat (http://tomcat.apache.org/), which is an open source software implementation of the Java Servlet and JavaServer Pages technologies and enables easy scalability by using load-balancing between multiple Tomcat servers. To enable secure communication to the GiraffPlus LTS web service, both the server and the client must present valid certificates issued by the GiraffPlus Certificate Agency, which is used to authenticate both parties and to encrypt all exchanged data. Data between the web service and its clients is exchanged in JSON format and, if required by the client, compressed to increase the efficiency of data transfer.

### 8.4. The GiraffPlus Engineer GUI

The GiraffPlus Engineer GUI enables the GiraffPlus professionals to enter and edit configuration data for any home included in the GiraffPlus ecosystem. The GUI is a wizard-like web application, which can be used from any web-enabled device using any the major web browsers. It was implemented in Java using the Play Framework (http://playframework.com/). In the back-end, it communicates securely with the GiraffPlus Storage Web Service to store all configuration data in the underlying database.

### 8.5. The GiraffPlus Certificate Agency

The GiraffPlus Certificate Agency was created due to the need for secure communication between various GiraffPlus components and to reliably authenticate end-users. It is implemented on a stand-alone secure server with a front-end web service implemented in Java using the open source BouncyCastle libraries (http://www.bouncycastle.org/).

## 9. Context Recognition and Configuration Planning

The responsibility of the context recognition service (CRS) within the GiraffPlus system is to produce timelines containing inferred activities from the collected sensor data and relay these to the visualization systems, which, in turn, can present them to authorized users. These timelines are produced “off-line” on demand, i.e., as a response to a query from a user who wants to view the activities of the primary user over time. Characteristic of this approach is the fact that it can take into account future data and use that to revise past inferences, which can be altered or extended as new data arrives, while an “on-line” approach, as the name suggests, continuously commits to and inferred activity for the current point in time which will not be revised in the future, even if presented with contradictory data. Another responsibility of the CRS is to raise alarms when specific conditions are met (e.g., the primary user has been staying in the bathroom for an abnormal amount of time, which might indicate a fall). Alarms are implemented by continuously querying the context recognition system at regular intervals (in this case, the system only takes into account past data to infer the presence of an alarm at a given point in time).

### 9.1. Context Recognition

The context recognition system represents the states of the sensors and the inferred activities as temporal intervals on different timelines. The model that describes the causal relationships between the states of the sensors and the inferred activities is provided as a set of quantitative Allen's interval algebra [14] constraints that are posted between intervals representing sensor readings. For instance, the rule, Cooking Equals Stove_OnΛCooking During In_Kitchen, defines how an activity, in this case, the activity Cooking, can be inferred from intervals representing sensed data (cooking implies the stove being on while the inhabitant is present in the kitchen). This approach to context recognition was first described by Ullberg *et al.* [15] and then subsequently extended by Pecora *et al.* [16]. However, in prior work, it was found that, although constraints taken from Allen's interval algebra provides a convenient way to describe relations in a flexible manner, small deviations in how the raw sensory data is interpreted and placed on the timelines can prevent activities from being inferred and, in some cases, produce false positives. One possible way of overcoming this is by allowing constraint violations to some degree; this can, for instance, be done by using fuzzy Allen's interval constraints [17]. However, this former approach requires thresholds on the likelihood of inferred activities, which is not always possible to determine. The context recognition in the GiraffPlus project handles the problem differently by performing temporal inference on multiple intervals contextually, admitting several interpretations of sensor and activity timelines. That is, each sensor reading is represented as a set of flexible temporal intervals rather than a single one. The former approach to context recognition is described in [18], where it is compared to the Chronicle recognition approach to context recognition proposed by Dousson and Maigat [19], in which Allen's interval constraints are also used.

The context recognition system is implemented as three separate modules; preprocessing, inference and extraction. The modules are implemented as RESTful web services that run on the central data storage server. Their responsibilities are as follows:

- ***Preprocessing*** The main responsibility of the preprocessing module is to fetch and process data from the data storage. This module can be called either from the inference module (or directly by the user in case a “simple activity” is being requested, such as the state of a door). The module implements an array of methods that can be applied to the data. The main characteristic of this module is that it only reason and processes data from one sensor or a set of similar sensors (for instance, all infrared sensors, to determine the location of the person in the environment) at a time. In addition, the preprocessing module caches frequently and recently accesses data in memory to reduce the load on the database server. The output of this module is a set of admissible sensory-timeline candidates.
- ***Inference*** The inference module uses inference rules to request sensory-timeline candidates from the preprocessing module based upon the activity that is being queried. Once all data has been supplied to this module, it infers a set of admissible activity-timelines by propagating constraints on the sensory-timeline candidates, the output is a set of possible activity timelines.
- ***Extraction*** The extraction module is used by the inference module to inspect the set of activity timelines and extracts a single timeline that can be used by the rest of the system. This module generates one single timeline from the set of activity-timeline candidates that are generated by the inference module's temporal propagation. The extracted timelines are visualized to a user or scanned for alarm conditions depending on the context in which the CRS is called.

**Figure 7.**
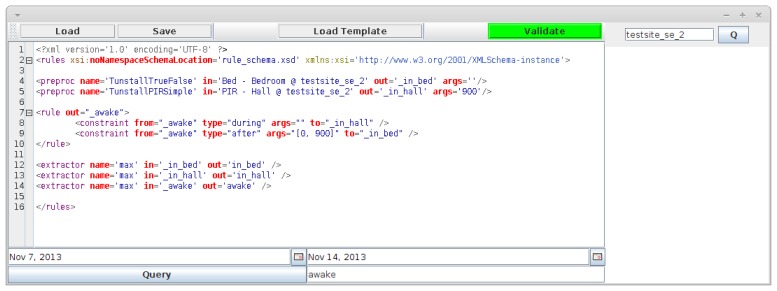
A rule editor that is used to create and send queries to the context recognition system.

As mentioned, the inference modules request data and timeline extraction from the preprocessing and extraction module. The way in which an activity is inferred is described in one or more rule files in XML format. An example of a simple rule file is shown in Figure 7; here, the preprocessing elements defines how data is treated (in this case, the data is processed using two functions called TunstallTrueFalse and TunstallPIRSimple, which determine how the raw sensory data should be interpreted).

**Figure 8.**
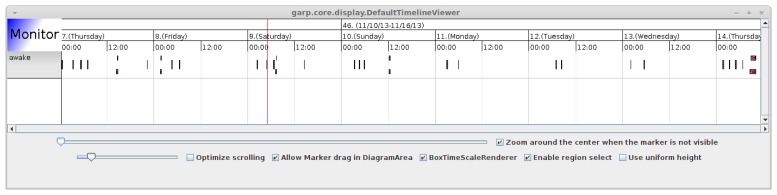
A timeline viewer showing the inference results from the query in Figure 8.

The file also defines the conditions under which an activity, _awake, should be inferred. Here, awake is defined as motion in the hall following a period of being in bed. The reason for including the sensor in the hall is that the person can trigger the motion sensor while sleeping; therefore, passing the hall is included to filter out these false positives. Finally, the extractor elements define the type of timeline that is requested; in this case, the elements define that we want to extract timelines where the intervals have the maximum possible temporal duration. The results of the inference can be seen in Figure 8, which shows time periods where the person is awake. This timeline was generated from real data coming from a Swedish test site and shows the time periods where the person has just woken up. As we can see, this (naturally) happens in the mornings, but also sometimes during the night.

## 10. Testing of the System in Real Homes

**Figure 9.**
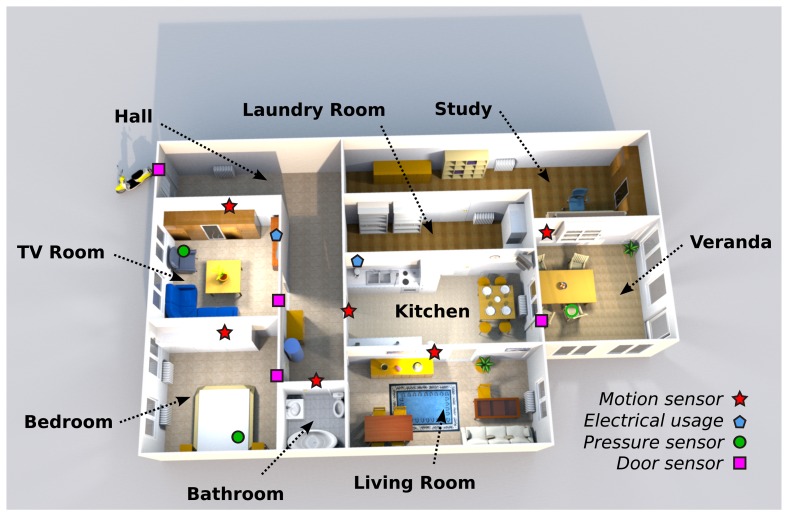
The map of one of the test site in Sweden showing the sensor positions.

The system is currently being tested in six real homes (two in Sweden, two in Spain and two in Italy), where the system has been deployed and will be used for one year. The system will be placed in nine additional homes in the spring of 2014. A map of one of the homes in Sweden with the positions of the sensors installed is shown in Figure 9. This particular apartment is inhabited by an 82-year-old man (born 1931) who has been living alone since his wife departed two years ago. He had a stroke two years ago and spends most of his time inside; the exceptions are when he goes outside to do shopping or to visit any of his three sons with his mobility scooter.

The sensors that are currently present in the apartment are the ones previously described. The Giraff robot present in the apartment is an updated prototype version of the robot equipped with a touch screen. As we previously stated, a key factor for the GiraffPlus system is the smooth, easy and reliable integration of all hardware components and the integration of the hardware components with the higher level of the system performing monitoring and reasoning on data. The test site was used to validate this aspect. Once the sensors are physically installed in the home, an operator simply configures them on the GiraffPlus Engineer GUI (Section 8.4), specifying the locations; then, the system automatically recognizes and exposes them through the middleware on the right topics. Henceforth, any application deployed in the home can gather and use sensed data.

### 10.1. Sensor Integration

**Figure 10.**
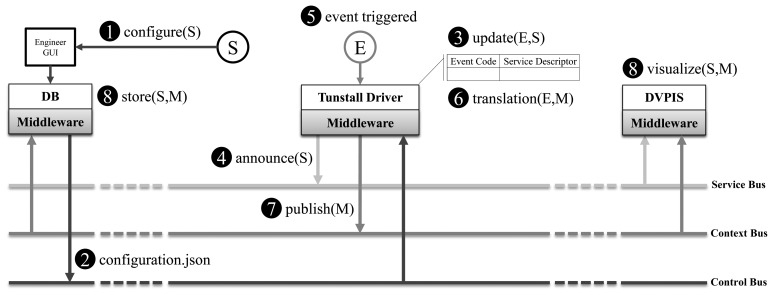
The sensor integration mechanism.

The integration of the sensors in the GiraffPlus system has been achieved by developing two connectors: one for the environmental sensors and one for the physiological sensors. Since the environmental sensors uses a gateway (the Lifeline Vi+) to collect all the events from the devices, a component that retrieves the configurations from the database, initializes the sensors and exposes it to the service bus has been implemented. The component, deployed to the set-top box installed in the primary user's house, listens for sensed data and publishes the information to the context bus when an event is raised. Figure 10 shows the steps involved in the integration of the Tunstall gateway and how its data format is translated into the data schema managed by the middleware (Section 6.3). When a new sensor is installed in the house and is configured by means of the Engineer GUI (Step 1), the server infrastructure middleware instance publishes on the control bus topic:

≪location≫/controlBus/configuration

a JSON file (configuration.json) containing the new configuration (Step 2). Since the Tunstall sensor data schema is composed of several event codes representing all the possible events that a sensor can trigger, the Tunstall driver component developed upon the GiraffPlus middleware maintains in memory a table that associates a possible event to a sensor, described in terms of *ServiceDescriptor*. This table is updated with the information about the new configuration forwarded by the middleware (Step 3), and an announce is called on the service bus, notifying all the interested services about the presence of a new sensor (Step 4). When an event is triggered by the new sensor (Step 5), a translation from event code to the GiraffPlus message format is done (Step 6), and the message is published on the context bus topic composed of the keyword “sensor” and the configured “id” (Step 7). Once the message is published, each component that was subscribed on that topic will receive the message (Step 8). This is the case of both DVPIS, monitoring real-time data, and the GiraffPlus MQTT Listener, storing historical context data for future long-term data analysis. These steps are also made when a sensor is removed or changed in its configuration.

The same mechanism can be used to integrate and interoperate with different kinds of sensors based on other communication protocols, like ZigBee [20]. In the case of physiological sensors, an Android-based tablet is used as a gateway running the version of the middleware infrastructure (Section 7). It is used by the gateway application to announce the installed sensors to the service bus and to publish events from devices to the context bus. The Android version of the system has proven useful, since it added new possibilities for multisensor data integration on the proposed AAL scenario, since any embedded sensor on a generic Android-based device can be plugged in the GiraffPlus system [21].

Another key aspect tested in the home, besides the adaptability to changes in sensor configuration, is the reliability over long periods of time. A failover mechanism on the communication connector has been implemented exploiting the middleware functionalities given by the control bus. There are two kind of failures that can interrupt the flow of data for long-term monitoring: a failure on the data storage system or a failure on the connection link from the house to the GiraffPlus LTS. In the first case, since a heartbeat signal is sent by the GiraffPlus LTS component on the control bus and each middleware instance monitors this control bus topic, when no heartbeat is detected, a local replica of the data is stored to be sent again when the remote component comes back alive. In the second case, when a connection loss is detected, a local dispatch queue is set up that stores data until the connection is restored.

### 10.2. Context Recognition System

A context recognition system (CRS) is currently available in test sites. The system is able to infer context from sensory data represented as intervals on different timelines. It retrieves sensor data from the data storage, and on the basis of these data, it infers which activities have been performed in the home. Given that the system does not identify specific persons, the recognition is based on the assumption that just one person is present in the home. Table 1 shows the activities that are recognized by the context recognition and which sensors are used.

Evaluating the capability of the CRS to recognize activities depends on our ability to compare inferred context with ground truth. However, gathering ground truth is difficult in our testbeds for two reasons. First, requiring the user to annotate the daily activities sporadically will inevitably lead to sparse data. Conversely, regularly prompting for annotations as done in [22] is deemed overly burdensome for our users. Second, privacy requirements prevent us from placing cameras in the environment so that the ground truth can be established by a third party. We will therefore focus on evaluating the perceived usability by the caregivers and end users of the overall system rather than directly focusing on metrics, like precision and recall. The fact that rules can be adapted for specific users and sensors facilitates deployment in heterogeneous environments. However, the heterogeneity of environments sometimes poses significant challenges that cannot be overcome by system configuration. For instance, a wall mount makes it impossible to monitor the usage of the microwave oven in one test site, while in another home, some appliances consume a high level of electricity, even in sleep mode, which makes it impossible to distinguish between the on and off state. Catering for these practical difficulties in the sensor design is a key enabler for success.

### 10.3. Data Storage

The system installed in the test homes has been running for about a year. In this year, the data storage component has logged 99,176 sensor data days from 14,870 distinct sensors. At the end of each day, the data collection of the previous day is compressed by removing all duplicated information posted by the sensors (e.g., the sensor ID is posted in each sensor reading, and it is not useful to store all samples with this string). The resulting sensor reading and timestamp arrays are compressed (gzipped) to save space. This makes queries a lot faster (a smaller number of entries in the sensor database) and saves space. Thanks to the nature of the sensor readings, publishing data only on status changes and when a measurement is made, the bandwidth assigned to sensor data can be limited.

During the first year of tests, the current system based on MongoDB has proven scalable. Its *sharding*feature has been used, which divides the database along a chosen key, where each shard is itself a replica set consisting of three or more replicas, which enabled us to gracefully handle any failures on the sets. Shards and replica sets may be geographically far apart, allowing clients to connect to the database in the most efficient way with respect to their network connectivity. Furthermore, the Mosquitto MQTT broker used, which can itself be distributed to different geographical locations and connected via “bridges” to each other, proved scalable. Regarding the RESTful GiraffPlus Storage Web Service, to scale up our ability to serve increasing numbers of web service users, an Apache proxy, in combination with a load balance able to distribute requests to multiple Tomcat servers, has been used. Since web services expose a REST interface (all services are stateless), we could scale up as needed.

## 11. Conclusions

In this paper, we presented GiraffPlus, an implemented system for supporting independent living for the elderly. The system includes many components, from sensors to high-level reasoning. A middleware is used to integrate them and to make the addition and removal of components easy. We identified the crucial features of the system in the easy and reliable integration of all the hardware and software components, the possibility of the quick installation of new hardware and software modules, the adaptability to make changes in the configuration and the reliability over long periods of time. These features have been tested in a real home environment. This is another key aspect of the proposed solution. In fact, the system has been tested and is currently deployed in real homes across Europe for the long-term monitoring of physiological data, the activities of daily living and the behaviors of elderly people.

## Figures and Tables

**Table 1.** Activities that can be recognized by the context recognition system.

**Activity**	**Used Sensors**
In bed	Pressure sensor underneath the mattress.
Position	Motion sensors placed in each room of the home, door usage for outdoor activities.
Cooking	Electrical usage sensor connected to the stove/microwave oven.
Watching TV	Electrical usage of the TV, motion sensors and pressure in the TV chair.
Awake at night	Bed pressure and motion sensor (see Figure 8).
